# Malabsorption Due to Chronic Giardiasis as a Presenting Symptom of Common Variable Immunodeficiency

**DOI:** 10.7759/cureus.12201

**Published:** 2020-12-21

**Authors:** Débora Sousa, Tiago Neto Gonçalves, Natália Marto, Alexandra Bayão Horta

**Affiliations:** 1 Internal Medicine Department, Hospital da Luz Lisboa, Lisbon, PRT

**Keywords:** common variable immunodeficiency, giardiasis, malabsorption, anemia

## Abstract

Common variable immunodeficiency (CVID) is a primary immunodeficiency that presents with a broad spectrum of clinical manifestations. We report the case of a 33-year-old man, initially referred to the outpatient internal medicine clinic for anemia. At the evaluation, the patient complained of diarrhea and unintentional weight loss for the last six months. He had no known medical conditions, but his previous medical history highlighted recurrent respiratory infections since childhood and also oral ulcers. The investigation identified iron-deficiency anemia caused by a malabsorption syndrome due to chronic giardiasis (Giardia lamblia cysts identified in fecal culture and Giardia lamblia trophozoites identified in the villi epithelium). Further investigation revealed bilateral bronchiectasis and splenomegaly. Suspecting CVID, a serum protein electrophoresis was performed, which showed a flattening of the gamma region, corresponding to a severe deficit of immunoglobulin (Ig) G, IgA, and IgM. A deficiency in the production of IgG in response to immunizations was confirmed, and the other causes of hypogammaglobulinemia were excluded. Therefore, a diagnosis of CVID was established. Malabsorption due to chronic giardiasis is a rare cause of iron deficiency anemia and an unusual presentation of CVID.

## Introduction

Common variable immunodeficiency (CVID) is a primary immunodeficiency disorder characterized by immune deregulations resulting in B-cell differentiation failure, with the impaired secretion of immunoglobulins [1]. It is characterized by a severe deficit of immunoglobulin (Ig) G, IgA, and IgM as well as a deficient or absent response to immunizations, after exclusion of other causes of hypogammaglobulinemia [2, 3].

This disease exhibits a broad range of clinical manifestations, including recurrent respiratory tract infections inducing chronic lung disease, liver and gastrointestinal disorders, and granulomatous disease. The immune dysregulation also contributes to autoimmune disorders, inflammatory diseases, and an increased risk of malignancies such as lymphomas and gastric cancers [2, 3].

CVID is a rare disorder; the incidence is estimated to be between 1:10 000 and 1:100 000 of the population. Due to its broad range of clinical presentations, the diagnosis is often delayed until adulthood, usually between the ages of 20 and 40. By this time, many patients already have irreversible consequences of the disease [1-4].

## Case presentation

We report the case of a 33-year-old man referred to the outpatient internal medicine clinic due to anemia. At admission, the patient complained of diarrhea and unintentional weight loss for the last six months. He described recurrent episodes of diarrhea, with mucus and pus, usually presenting with more than 10 episodes a day, including nighttime, associated with abdominal pain. These episodes would last five days and then resolve spontaneously and recur approximately two weeks later. He had severe anorexia and lost seven kilograms (10% body weight) during this period.

He had no known medical conditions, but his previous medical history highlighted recurrent respiratory infections since childhood and also frequent oral ulcers. He didn’t take any medication, and in his family, there were no known hereditary immunodeficiencies or autoimmune diseases. He hadn’t traveled to any foreign country in the previous five years.

Physical examination revealed remarkable pallor and slight splenomegaly, and blood tests depicted microcytic anemia (hemoglobin of 9.7g/dL, the mean corpuscular volume of 69fL), with absolute iron deficiency (ferritin of 12ug/L).

**Figure 1:**
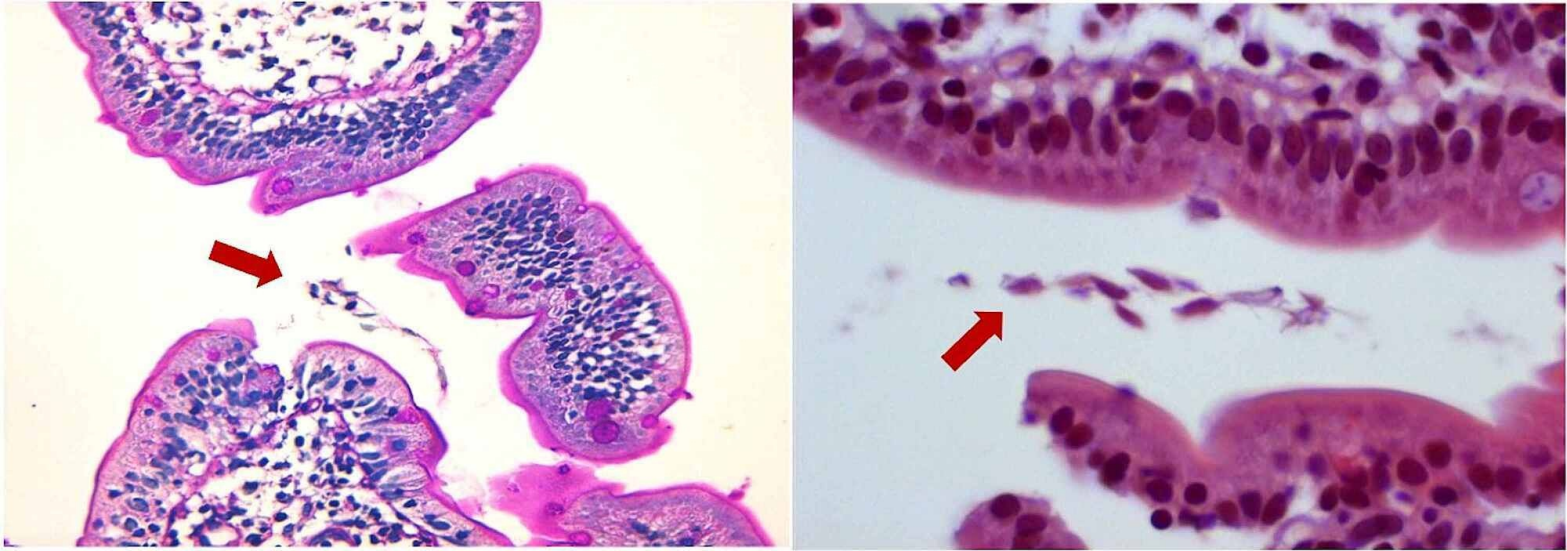
Giardia lamblia trophozoites (red arrows) along the surface of the villi epithelium

Investigations proceeded with feces cultures, which isolated Giardia lamblia cysts. Upper endoscopy and colonoscopy showed normal gastric and intestinal mucosa, but duodenal biopsies revealed an inflammatory lymphocyte infiltrate in the lamina propria, the tell-tale absence of plasmocytes, and the presence of Giardia lamblia trophozoites (tear-drop shaped) in the villi epithelium (Figure 1). 

Computed tomography (CT) of the thorax and abdomen showed bilateral bronchiectasis and splenomegaly.

**Figure 2:**
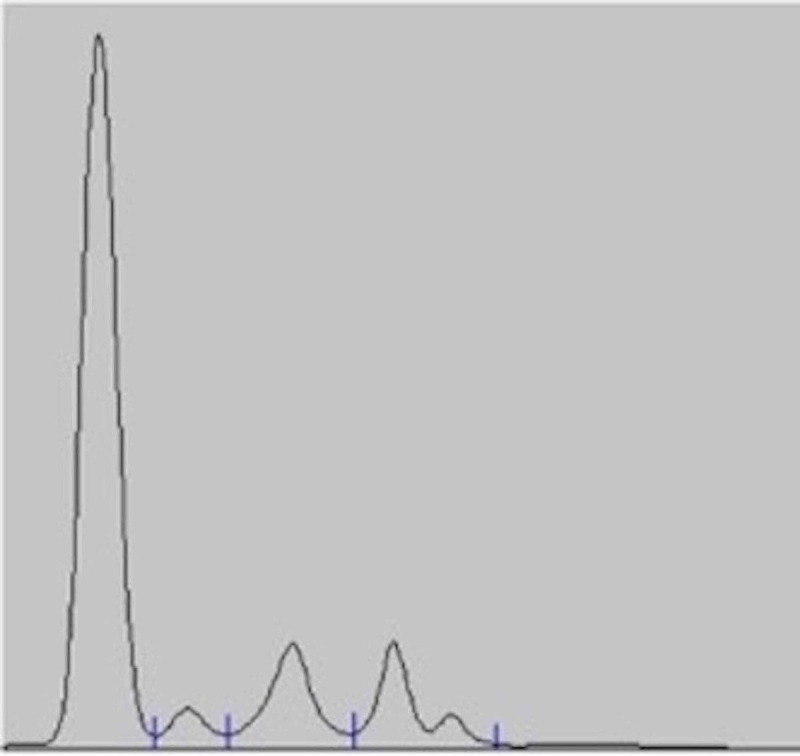
Serum protein electrophoresis showing a marked flattening of the gamma region

In view of these findings, a suspicion of CVID arose. The serum protein electrophoresis showed a marked flattening of the gamma region (Figure 2), and the measurements of the immunoglobulins showed he had a severe deficit of IgG (73mg/dL), IgA (undetectable), and IgM (15mg/dL). Additionally, there was a deficiency in the production of IgG in response to immunizations (tetanus and pneumococcal vaccines). 

We found no evidence of other causes of immunodeficiency. The viral serologies for the human immunodeficiency virus 1 and 2, hepatitis B virus, and hepatitis C virus were negative; he had no evidence of nephrotic syndrome; the CT scan didn’t find any solid lesions suggestive of cancer, and he also had no evidence of thymoma (excluding Good's syndrome). 

A diagnosis of CVID and chronic giardiasis was established, and the patient was treated with metronidazole with the improvement of the gastrointestinal symptoms. He started regular treatment with intravenous immunoglobulin (every four weeks), and at a ten-year follow-up, he is doing well.

## Discussion

Although rare, CVID is the most frequent symptomatic primary immunodeficiency. It is a diagnosis of exclusion that requires a high degree of suspicion [1, 2]. Early diagnosis of CVID can prevent significant morbidity and mortality. If started early, treatment with intravenous immunoglobulin can keep patients free of infections and minimize the effects of chronic and irreversible conditions, such as chronic pulmonary disease [4].

In the case reported, the presenting symptom that led to the diagnosis of CVID was anemia, which we now believe was due to malabsorption as a result of chronic giardiasis.

Some European reports have found some form of enteropathy in 9% of the CVID patients, but approximately 20% exhibit a multitude of gastrointestinal symptoms [1]. 

Giardia lamblia is a frequent culprit of acute gastrointestinal tract infection, especially in infants and in developing countries, but the chronic infection is rare. The manifestations of chronic giardiasis may wax and wane over many months, just like in our patient [5]. Giardiasis causes noninvasive colonization of the upper part of the small intestine: duodenum and jejunum. The pathogenic action of the protozoan and the inflammatory response activated in the host cause damage to the absorptive mucosa, with disruption of innate mucosal protective barriers. This results in malabsorption triggering symptoms such as diarrhea, abdominal pain, anorexia, and weight loss. As a result, patients will have many vitamin and mineral deficits such as vitamin A, thiamine, vitamin B12, folic acid, and iron, resulting in anemia [5].

## Conclusions

This case highlights that despite CVID being an infrequent entity, clinicians should have a high degree of suspicion for the diagnosis of CVID in patients with recurrent respiratory infections and oral ulcers. It is also important to recognize that pathogens that usually don't cause chronic diseases, such as Giardia, should raise the hypothesis of CVID.
